# Genome Sequences of *Listeria monocytogenes* Strains Responsible for Cheese- and Cooked Ham Product-Associated Swiss Listeriosis Outbreaks in 2005 and 2011

**DOI:** 10.1128/genomeA.00106-16

**Published:** 2016-03-10

**Authors:** Taurai Tasara, Jochen Klumpp, Jacques Bille, Roger Stephan

**Affiliations:** a Vetsuisse Faculty, Institute for Food Safety and Hygiene, University of Zurich, Zurich, Switzerland; b Institute of Food, Nutrition and Health, ETH Zurich, Zurich, Switzerland; c Institute of Microbiology, University of Lausanne, Lausanne, Switzerland

## Abstract

The complete genome sequences of three *Listeria monocytogenes* serotype 1/2a strains, Lm 3136, Lm 3163, and Lm N1546, which were responsible for listeriosis outbreaks in 2005 and 2011 in Switzerland, are presented here.

## GENOME ANNOUNCEMENT

Listeriosis is a severe life-threatening illness that can lead to high mortality among those with weakened immune systems caused by *Listeria monocytogenes* (1). In 2005 and 2011, Switzerland experienced two listeriosis outbreaks linked to the consumption of soft cheese and imported cooked ham products, respectively, which were contaminated with serotype 1/2a *L. monocytogenes* strains (2, 3). The genome sequences of three *L. monocytogenes* strains responsible for these outbreaks were determined. Strains Lm 3136 and Lm 3163 are both clinical isolates with different pulsed-field gel electrophoresis (PFGE) pulsotypes isolated from patients during the 2005 Tomme cheese outbreak (2). Strain Lm N1546 is a patient isolate recovered during the 2011 outbreak linked to contaminated imported cooked ham products (3).

Genomic DNA isolated from brain heart infusion (BHI) agar-grown cultures of all three strains using the Sigma genomic DNA kit were sequenced using the single-molecule real-time sequencing technology on a Pacific Biosciences RSII device (10-kb insert library, P6/C4 chemistry) at the Functional Genomics Centre Zurich (FGCZ). Sequencing generated 62,200 reads averaging 5,258 bp in length for Lm 3136, 42,920 reads averaging 8,903 bp for Lm 3163, and 56,797 reads averaging 8,631 bp for Lm N1546. Using the SMRT Analysis 2.3.0 software, the Lm 3136 and Lm 3163 genomes were *de novo* assembled into single chromosomes of 2,905,347 bp and 2,927,751 bp in size, respectively. The Lm N1546 genome was assembled to a chromosome of 2,952,608 bp and a plasmid (pLmN1546) of 86,616 bp in size. All three genomes were annotated using the NCBI Prokaryotic Genome Annotation Pipeline (http://www.ncbi.nlm.nih.gov/genome/annotation_prok/) (4).

The Lm 3136 genome contains 2,916 genes, 23 pseudogenes, and 67 tRNAs, whereas the Lm 3163 genome contains 2,922 genes, 11 pseudogenes, and 67 tRNAs. The Lm N1546 genome harbors 3,065 genes, 24 pseudogenes, and 67 tRNAs. Using the Phage search tool (PHAST) (5), two prophage regions each in Lm 3136 and Lm 3136, as well as three prophage regions in Lm N1546, were predicted. Lm 3136 contains one incomplete prophage (positions 2375273 to 2398241) and one phage-like region (positions 1671557 to 1720815), and Lm 3163 possesses one intact (positions 1932637 to 1977442) and one incomplete (positions 1387687 to 1410661) prophage, while Lm N1546 is predicted to harbor one intact (positions 2742105 to 2785466) and one incomplete (positions 1618930 to 1641905) prophage, as well as one phage-like region (positions 924531 to 963457). Lm 3136, Lm 3163, and Lm N1546 were assigned to sequence type 18 (ST18), ST26, and ST8, which were grouped into clonal complex 18 (CC18), CC26, and CC8, respectively, using multilocus sequence typing (MLST) analysis (http://www.pasteur.fr/recherche/genopole/PF8/mlst/index.html) (6). The genome sequences of these strains will be used for comparative analysis with those of other sequenced *L. monocytogenes* strains. Such comparisons will provide insights into genes underlying relevant virulence and stress resistance properties in this bacterium.

### Nucleotide sequence accession numbers.

The three complete genomes and the pLN1546 plasmid sequence are available in GenBank under accession numbers CP013722, CP013723, CP013724, and CP013725.
